# Post-Total-Pancreatectomy-Hemorrhage (PTPH) – approaching a new definition

**DOI:** 10.1007/s00423-025-03869-4

**Published:** 2025-09-23

**Authors:** Lea Timmermann, Richard Schensar, Federico Storni, Johann Pratschke, Thomas Malinka

**Affiliations:** Department of Surgery, Berlin Institute of Health, Charité – Universitätsmedizin Berlin, Campus Virchow-Klinikum, corporate member of Freie Universität Berlin, Humboldt-Universität zu Berlin, Augustenburger Platz 1, Berlin, 13353 Germany

**Keywords:** Total pancreatectomy, Postoperative bleeding, PTPH, PPH

## Abstract

**Purpose:**

Postoperative hemorrhagic complications in pancreatic surgery are classified according to the International Study Group for Pancreatic Surgery (ISGPS). However, following total pancreatectomy, the predominant bleeding causes associated with pancreatic fistula or insufficiency of the pancreatico-enteric anastomosis are eliminated. The objective of this study is to examine bleeding sources following total pancreatectomy, and propose a novel classification, termed Post-Total Pancreatectomy Hemorrhage (PTPH).

**Methods:**

An overall of 195 patients was included and reviewed for baseline characteristics, comorbidities, intraoperative findings, perioperative coagulation profiles, and postoperative courses. Applicability of the ISGPS classification to PTPH was critically assessed with respect to the existing criteria: timely onset, location and cause, and severity. Subgroups were defined with regard to bleeding sources including erosion, surgical, gastrointestinal, and diffuse bleeding. Furthermore, we developed a severity index to enhance objectivity.

**Results:**

Thirty-five of the patients experienced hemorrhagic complications. Timely onset and our severity index corresponded significantly with the bleeding source.

**Conclusion:**

The ISGPS classification, although widely utilized in pancreatic surgery, does not fully account for the bleeding complications associated with total pancreatectomy. Our proposed classification for PTPH introduces a more granular and clinically relevant framework, with clearly delineated subgroups based on source, and an innovative severity index.

**Supplementary Information:**

The online version contains supplementary material available at 10.1007/s00423-025-03869-4.

## Introduction

In the early years of pancreatic surgery, surgeons sought to avoid performing total pancreatectomies due to their profound endocrine and exocrine consequences. However, research has increasingly validated the safety and feasibility of this procedure, with studies demonstrating favourable short- and long-term outcomes [1, 2], a comparable quality of life [3], and a gradual reduction in associated morbidity and mortality [4]. This decline in adverse outcomes has fostered enthusiasm, as it obviates the need for the complex and high-risk pancreatico-enteric anastomosis inherent to organ-preserving techniques. Additionally, the rate of multifocal carcinoma unseen in the preoperative imaging is a discussable aspect [5]. Nonetheless, total pancreatectomies continue to be associated with significant morbidity and mortality, including bleeding complications.

Bleeding complications in the postoperative course of pancreatic surgery are currently classified as postoperative pancreatic haemorrhage (PPH) according to the guidelines established by the International Study Group of Pancreatic Surgery (ISGPS) [6]. Among the various aetiologies, pseudoaneurysms and erosions of visceral vessels - conditions strongly associated with postoperative pancreatic fistula (POPF) [7] or insufficiency of the pancreatico-enteric anastomosis- represent the predominant causes of bleeding in pancreatic surgery [8]. Following a total pancreatectomy, the likelihood of these bleeding sources is diminished, as the absence of pancreatic tissue precludes the development of POPF.

This study, unique in its focus on total pancreatectomy, examined the bleeding incidence, timely onsets, and bleeding sources following this procedure. The ISGPS definition, based on a triad of criteria: timely onset, location, and cause and severity, was assessed regarding its applicability. However, we developed a new classification system specific to total pancreatectomy. By comparing clinical parameters between patients experiencing bleeding complications and those without and by categorizing subgroups according to the source of bleeding, we seek to enhance our understanding of this complication entity. These findings will undoubtedly influence future research and clinical practice, providing a deeper understanding of post-total-pancreatectomy hemorrhage.

## Methods

Our prospective database identified and further reviewed all consecutive patients undergoing total pancreatectomy from January 2013 to July 2022 in our tertial referral center for pancreatic surgery. Conduction of this retrospective study was approved by Charité ethics committee (EA1/145/23).

Patients undergoing total pancreatectomy following pancreas transplantation were excluded from this study due to the unique nature of their surgical history and potential impact on bleeding complications. All patients undergoing another procedure to the pancreas than mentioned above (e.g., partial pancreaticoduodenectomy, distal pancreatectomy, or enucleation) were also excluded from further analysis as were patients under age, to ensure a homogeneous study population. These exclusion criteria were chosen to ensure that the study focused specifically on total pancreatectomy and its associated bleeding complications, providing a more accurate and applicable understanding of this specific complication. All bleeding complications in from the initial inhospital stay or readmission within 90 days after surger were included.

### Preoperative assessment and postoperative course

The preoperative assessment was standardized, encompassing a range of evaluations, including laboratory tests, physical examinations, and anaesthesiological risk stratification. Abdominal imaging was performed to ascertain local resectability using computed tomography (CT) or magnetic resonance imaging (MRI). If necessary, additional staging procedures, such as chest imaging or endosonography, were undertaken to provide a comprehensive assessment. This thorough evaluation of the oncological considerations facilitated a well-informed decision-making process regarding the surgical intervention. Patients were transferred to our surgical ward after an initial stay at our intensive care unit. All patients were provided with proton pump inhibitors (PPI) in the postoperative course and after discharge, ensuring the highest standard of care. Laboratory testing were performed in a routine setting. In Germany, the prothrombin time is commonly expressed as Quick value (%) and not in seconds. A low Quick value (PT in %) corresponds to a prolonged prothrombin time (PT) in seconds, indicating an inverse relationship between the two measurements.

### Surgical technique

Total pancreatectomy in our cohort was either performed in an open or robotic-assisted approach. For open surgery, an oblique incision was made, and extra pancreatic metastases were ruled out macroscopically, followed by local exploration confirming resectability. The omental bursa is opened, followed by Kocher’s manoeuvre. The pancreas is then dissected on the mesenterico-portal axis with a monopolar device, and both the head and tail are removed through sharp dissection. In the case of pancreatic head malignancy or no suspected malignancy, preservation of the spleen was attempted. However, the spleen was removed if chronic pancreatitis precluded separation of the pancreatic parenchyma from the splenic vessels or if intraoperative bleeding necessitated its removal, aside from oncological considerations. Hepaticojejunostomy is established using 5 × 0 PDS with a continuous technique for the dorsal portion and single sutures for the ventral part. Gastrojejunostomy is established with a double-row continuous suture using 4 × 0 PDS.

For minimally invasive procedures, in our cohort, only robotic-assisted cases, trocars were placed according to our standard operating procedure (SOP) [9]. Diagnostic laparoscopy rules out extra pancreatic spread. The remainder of the procedure followed a similar approach to the open technique, with sealing devices employed for tissue preparation and monothermy used for parenchymal dissection. Hepaticojejunostomy is established using 4 × 0 V-Loc as a continuous technique for the anastomosis’ dorsal and ventral parts.

### Definition of bleeding sources

According to the ISGPS definition, the site or source of a bleeding complication determines its classification as PPH A, B, or C. We aimed to further identify factors influencing bleeding complications following total pancreatectomy in general and its specific entities. Unlike in PPH, lipase-induced vessel erosion is precluded following total pancreatectomy. We, however, classified our entire cohort into four distinct bleeding entities or sources. The first entity encompasses bleeding resulting from erosion, which is attributed to insufficiencies in hepaticojejunostomy, gastrojejunostomy, or intra-abdominal abscesses. The second entity refers to surgical bleeding originating from a circumscribed vessel, typically due to intraoperative injury or inadequate ligation. The third entity is characterized by a lack of definite source such as a certain vessel in a surgical bleeding. In our cohort, we defined the group of diffuse bleeding as the (transient) intraabdominal and extraluminal bleeding, where CT-scans ruled out a certain bleeding source. An example is a markedly increased sanguineous output via the intra-abdominal drains or the onset of a relevant intraabdominal haematoma requiring treatment such as coagulation management or blood transfusion. The fourth entity includes gastrointestinal (GI) bleeding.

### Definition of severity

Following the ISGPS definition, we developed a scoring system designed to enhance the objectivity in assessing the severity of bleeding, expressed as interval-scaled data. This new scoring system represents a substantial advancement in the field, providing a more nuanced and comprehensive understanding of the severity of bleeding. The severity of bleeding was assessed based on the occurrence of organ failure, the requirement for ICU admission or intervention, and the necessity for blood transfusion.

The following table outlines the scoring system, providing a clear and comprehensive understanding of how the severity of bleeding was assessed. (Table 1). The aim of the severity index is to enhance inter-institutional comparison and research in the field of post-pancreatectomy-hemorrhaghe.

**Table 1:** Score for severity of bleeding

	Score	Notes
Organ failure	0 = no organ failure 1 = one organ failure 2 = multi organ failure 3 = CPR	
ICU-admission	0 = no 1 = yes	Score 1, if patient is already/still on ICU
Intervention	0 = none/observation 1 = intervention (e.g. EGD/angiography) 2 = surgery	
Necessity for blood transfusion	0 = no 1 = yes	
	Total: 7 points	Immediate death equals 7 points as the score system in this point underestimates severity

CPR: cardiopulmonary resuscitation

### Statistics

Data were processed using SPSS version 29.0 (IBM, Armonk, NY, USA). Two-tailed Pearson’s chi-square test and Mann-Whitney-U-test were performed on categorical and ordinal scaled data, and student’s t-test was performed on interval scaled data. Significance tests were two-sided, and p < 0.05 was considered statistically significant. A subgroup analysis was performed for cases without insufficiency to the hepaticojejunostomy using two-tailed Pearson’s chi-square test, Mann-Whitney-U-test on categorial and ordinal scaled data, and student t-test on interval scaled data. Kruskal-Wallis-test was performed on categorially scaled data with n > 2 categories.

Successive backward and forward bivariate and multivariate logistic regression was applied to the whole data set to examine predictive patterns or combinational risk factors.

## Results

195 patients were included. Thirty-five of them developed bleeding complications.

### Baseline characteristics and patient’s history

Baseline characteristics are comparable in both groups. Tumor grading exhibited a statistically significant difference, with a higher grade observed in the group without bleeding complications (p = 0.03). There was also a notable difference in the L stage, with increased lymph vessel invasion identified in the group experiencing bleeding complications (p = 0.025). Additionally, the V stage differed statistically significant, revealing more vessel invasion among patients with bleeding complications (p = 0.025).

Table 2. indicates the patient’s demographics.

**Table 2:** Patient’s demographics

	All patients (n = 195)	Bleeding complications (N = 35)	No bleeding complications (N = 160)	p-value
Age (years)				1.0
Minimum	34	38	34	
Maximum	86	82	86	
Median	67	68	67	
Sex N (%)				0.407
Female	79 (41)	12 (34)	67 (42)	
Male	116 (60)	23 (66)	93 (58)	
BMI (kg/m²)				0.626
Minimum	17.2	17.7	17.2	
Maximum	42.8	38.6	42.8	
Median	24.6	24.15	24.8	
ASA classification N (%)				0.335
1	4 (2)	1 (3)	3 (2)	
2	64 (35)	8 (25)	56 (37)	
3	111 (60)	22 (69)	89 (58)	
4	6 (3)	1 (3)	5 (3)	
Malignancy N (%)	151 (77)	25 (71)	126 (79)	0.348
Diagnosis N (%)				0.405
PDAC	128 (66)	20 (57)	108 (68)	
Cholangiocarcinoma	10 (5)	1 (3)	9 (6)	
NET	7 (4)	3 (9)	4 (3)	
Chronic pancreatitis	24 (12)	5 (14)	19 (12)	
IPMN	17 (9)	5 (14)	12 (8)	
Other malignant	6 (3)	1 (3)	5 (3)	
Other benign	3 (2)	0 (0)	3 (2)	
NAC in cases of underlying malignancy N (%)	22 (14)	3 (12)	19 (14)	0.740
T state N (%)				0.054
1	15 (10)	4 (17)	11 (9)	
2	47 (32)	5 (22)	42 (34)	
3	77 (53)	11 (48)	66 (54)	
4	6 (4)	3 (13)	3 (3)	
N state N (%)				0.432
0	50 (35)	8 (35)	42 (34)	
1	63 (43)	7 (30)	56 (46)	
2	32 (22)	8 (35)	24 (20)	
Grading G (%)				0.03
1	7 (6)	2 (10)	5 (5)	
2	74 (59)	15 (75)	59 (56)	
3	45 (36)	3 (15)	42 (40)	
Resection state N (%)				0.492
0	88 (63)	13 (57)	75 (64)	
1	52 (37)	10 (44)	42 (36)	
L state N (%)				0.025
0	105 (75)	13 (57)	92 (79)	
1	35 (25)	10 (44)	25 (24)	
V state N (%)				0.025
0	123 (88)	17 (74)	106 (91)	
1	17 (12)	6 (26)	11 (9)	
Tumour size (mm)				0.376
Minimum	4	7	4	
Maximum	110	110	85	
Median	31	35	30.5	

BMI: body mass index; ASA: American Society of Anesthesiologists; PDAC: pancreatic ductal adenocarcinoma; IPMN: intraductal papillary mucinous neoplasm; NAC: neoadjuvant chemotherapy

No statistically significant differences were observed between the two groups concerning preexisting medical conditions. This includes factors such as preoperative.

anticoagulation, a history of bleeding, liver diseases, or alcohol abuse. Table 3. indicates the patient’s history.

**Table 3:** Patient’s history

	All patients (n = 195)	Bleeding complications (N = 35)	No bleeding complications (N = 160)	p-value
Insulin dependent diabetes mellitus N (%)	51 (26)	12 (34)	39 (24)	0.227
Medication for arterial hypertonus N (%)	103 (53)	22 (63)	1 (51)	0.189
History of thrombosis/embolism N (%)				0.237
Venous thrombosis	6 (3)	0 (0)	6 (4)	
Lung artery embolism	6 (3)	0 (0)	6 (4)	
Coronary heart disease/PAD	14 (7)	3 (9)	11 (7)	
Ischemic stroke	4 (2)	2 (6)	2 (1)	
History of bleeding N (%)	4 (2)	0 (0)	4 (3)	0.345
History of liver disease N (%)	13 (7)	2 (6)	11 (7)	0.803
History of alcohol abuse N (%)	20 (10)	3 (9)	17 (11)	0.717
Preoperative anticoagulation N (%)				0.501
Platelet aggregation inhibitor	23 (12)	4 (11)	19 (12)	
Coumarine derivatives	3 (2)	1 (3)	2 (1)	
Other oral anticoagulation	6 (3)	1 (3)	5 (3)	
Other	4 (2)	2 (6)	2 (1)	

PAD: peripheral artery disease

### Surgical findings

Although the median surgical time was longer in the group with bleeding complications, this finding did not appear to be statistically significant (p = 0.116). The performance of splenectomy also did not statistically significant influence the occurrence of bleeding complications (p = 0.723). The group with vascular resections was small, however, differences did not appear to be statistically significant (p = 0.501). The amount of administered intraoperative blood transfusions (p = 0.102) and fresh frozen plasma (p = 0.084) was higher in the group with bleeding complications, although these findings were not statistically significant. Table 4 presents the intraoperative and surgical findings.

**Table 4:** Intraoperative and surgical findings

	All patients (n = 195)	Bleeding complications (N = 35)	No bleeding complications (N = 160)	p-value
Surgical time (minutes)				0.116
Minimum	113	196	113	
Maximum	828	613	828	
Median	349	360	340.5	
Access N (%)				0.684
Open	184 (94)	34 (97)	150 (94)	
Robotic assisted	9 (5)	1 (3)	8 (5)	
Hybrid	2 (1)	0 (0)	2 (1)	
Splenectomy N (%)	95 (49)	18 (51)	77 (48)	0.723
Portal vein resection/reconstruction N (%)	15 (8)	2 (6)	13 (8)	0.628
Other vascular resections/reconstructions	10 (5)	1 (3)	9 (6)	0.501
Intraoperative blood transfusion (bags)				0.102
Minimum	0	0	0	
Maximum	11	11	8	
Median	0	0	0	
Intraoperative fresh frozen plasma (bags)				0.084
Minimum	0	0	0	
Maximum	29	29	22	
Median	0	0	0	

### Laboratory testing

The postoperative prothrombin time (%) was lower in the group with bleeding complications (p = 0.029). In contrast, the difference in preoperative prothrombin time (p = 0.447) and the prothrombin time on day one (p = 0.483), three (p = 0.345), and five (p = 0.223) after surgery did not reach statistical significance. The activated partial thromboplastin time on day three after surgery was statistically significantly longer in the group with bleeding complications (p = 0.007). Although the activated partial thromboplastin time preoperatively (p = 0.932), postoperatively (p = 0.102), and on day one (p = 0.105) and five (p = 0.427) after surgery was shorter in the group with bleeding complications, these findings did not appear to be statistically significant. The platelet count was significantly lower postoperatively (p = 0.027), one day after surgery (p = 0.001), on day three after surgery (p = 0.005), and on day five after surgery (p = 0.005) in the group with bleeding complications. Table 5 shows the laboratory testing.

**Table 5:** Laboratory testing

		Bleeding complications (N = 35)	No bleeding complications (N = 160)	p-value
Preoperative prothrombin time (%)				0.447
Minimum	58	66	58	
Maximum	128	120	128	
Median	95	91.5	96	
Postoperative prothrombin time (%)				0.029
Minimum	45	45	49	
Maximum	111	100	111	
Median	82	78	83	
Prothrombin time POD 1 (%)				0.483
Minimum	22	35	11	
Maximum	108	87	108	
Median	70.5	70	71	
Prothrombin time POD 3 (%)				0.345
Minimum	19	44	19	
Maximum	127	103	127	
Median	72	69	73	
Prothrombin time POD 5 (%)				0.223
Minimum	38	50	38	
Maximum	111	100	111	
Median	76	73	77	
Preoperative activated partial thromboplastin time (sec)				0.932
Minimum	23.2	25.5	23.2	
Maximum	70.5	58.4	70.5	
Median	33	32.7	33.1	
Postoperative activated partial thromboplastin time (sec)				0.102
Minimum	20.5	26.6	20.5	
Maximum	207.4	207.4	54.4	
Median	31	32.3	30.7	
Activated partial thromboplastin time POD 1 (sec)				0.105
Minimum	21.7	29.7	21.7	
Maximum	121.5	121.5	78.1	
Median	37.7	39.5	37.6	
Activated partial thromboplastin time POD 3 (sec)				0.007
Minimum	21.2	31.5	21.2	
Maximum	164.7	164.7	85.2	
Median	38.9	42.3	38.35	
Activated partial thromboplastin time POD 5 (sec)				0.427
Minimum	21	28.6	21	
Maximum	246	66.9	246	
Median	34.4	36.7	34.1	
Preoperative platelet count (/nl)				0.867
Minimum	109	116	109	
Maximum	674	545	674	
Median	248	247	248	
Postoperative platelet count (/nl)				0.027
Minimum	19	19	57	
Maximum	629	448	629	
Median	203	175	210	
Platelet count POD 1 (/nl)				0.001
Minimum	51	51	62	
Maximum	516	369	516	
Median	190.5	141	200	
Platelet count POD 3 (/nl)				0.005
Minimum	30	30	64	
Maximum	687	385	687	
Median	185	133	197	
Platelet count POD 5 (/nl)				0.005
Minimum	26	26	67	
Maximum	907	608	907	
Median	252	164	262	
Postoperative antithrombin 3 (%)				0.876
Minimum	45	51	45	
Maximum	119	119	119	
Median	81	81	81	
Antithrombin 3 POD 1 (%)				0.965
Minimum	6	57	6	
Maximum	120	110	120	
Median	81	79	81	
Postoperative fibrinogen (g/l)				0.102
Minimum	1.72	1.72	1.86	
Maximum	6.42	6.02	6.42	
Median	3.56	3.16	3.66	
Preoperative CA 19.9 (kU/l)				0.692
Minimum	1.7	2	1.7	
Maximum	6582	6582	5711	
Median	132.5	13.4	146	
Preoperative CEA (µg/l)				0.673
Minimum	0.7	2.2	0.7	
Maximum	78.7	22.5	78.7	
Median	3.2	5.8	3.15	

### Perioperative course and postoperative complications

Patients in the group with bleeding complications had a significantly longer inhospital stay (p = 0.005) and stay on the intensive care unit (p = 0.014). The rate of kidney (p < 0.001) and liver failure (p < 0.001) was significantly higher in the group with bleeding complications. The rate of insufficiency of the hepaticojejunostomy was significantly higher in the group with bleeding complications (p = 0.036). The reoperation rate (p < 0.001) was also significantly higher in the group with bleeding complications, as well as the 30-day mortality (p = 0.007) and readmission rate (p = 0.007). The occurrence of thrombosis or embolism was also significantly higher in the group with bleeding complications (p < 0.001). Table 6 indicates the perioperative course and postoperative complications.

**Table 6:** Perioperative course and postoperative complications

	All patients (n = 195)	Bleeding complications (N = 35)	No bleeding complications (N = 160)	p-value
Inhospital stay (d)				0.005
Minimum	1	4	1	
Maximum	217	217	88	
Median	17	26	15.5	
ICU stay (d)				0.014
Minimum	1	2	0	
Maximum	203	105	203	
Median	4	11	4	
Overall complications N (%)	127 (65)	35 (100)	92 (58)	< 0.001
Kidney failure	37 (19)	16 (46)	21 (13)	< 0.001
Liver failure	18 (9)	10 (29)	8 (5)	< 0.001
Insufficiency hepaticojejunostomy N (%)	20 (10)	7 (20)	13 (8)	0.036
SSI N (%)	38 (20)	10 (29)	28 (18)	0.134
DGE N (%)	27 (14)	4 (11)	23 (12)	0.648
Reoperation N (%)	42 (22)	22 (63)	20 (13)	< 0.001
30-day-mortaility N (%)	10 (5)	5 (14)	5 (3)	0.007
Readmission rate N (%)	24 (13)	9 (27)	15 (9)	0.007
Clavien/Dindo N (%)				< 0.001
1	5 (2.6)	1 (2.9)	4 (2.5)	
2	39 (20)	2 (5.7)	37 (23.1)	
3a	14 (7.2)	6 (17.1)	8 (5)	
3b	29 (14.9)	8 (22.9)	21 (13.1)	
4a	17 (8.7)	5 (14.3)	12 (7.5)	
4b	4 (2.1)	3 (8.6)	1 (0.6)	
5	21 (10.8)	10 (28.6)	11 (6.9)	
Thrombosis/embolism during inhospital stay N (%)	19 (9.7)	9 (25.7)	10 (6.3)	< 0.001

ICU: intensive care unit; SSI: surgical site infections; DGE: delayed gastric emptying

### Subgroup analysis excluding cases with insufficiency of the hepaticojejunostomy

We furthermore performed a subgroup analysis for all patients without an insufficiency of the hepaticojejunostomy. This analysis included an overall of 175 cases, of which 28 patients experienced bleeding complications over the course, and 147 did not. The supplementary files of this article provide tables related to this subgroup analysis (compare Supp. files Tab. 1.1-1.5).

### Bleeding source and severity

**Fig. 1:**
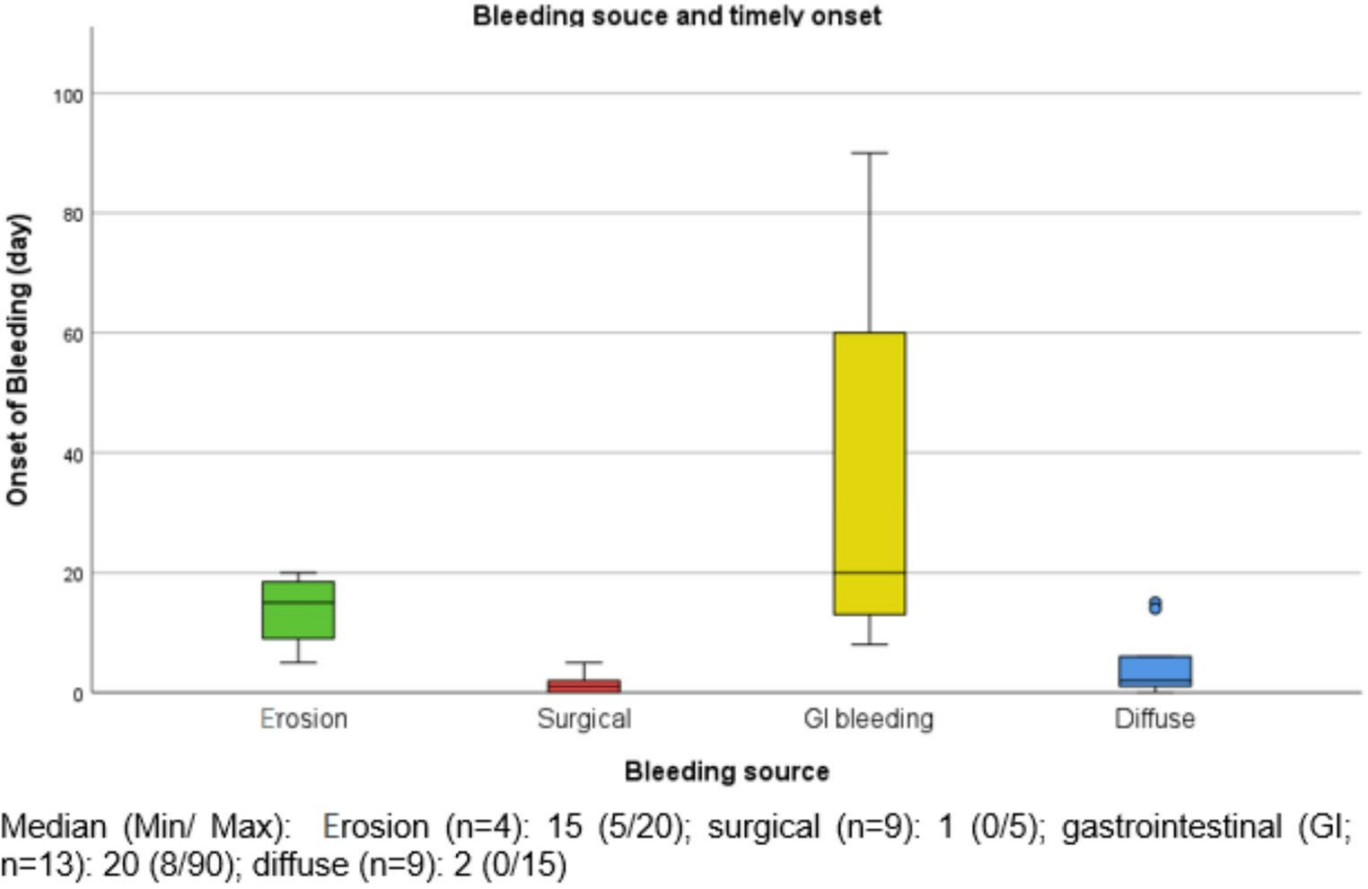
Bleeding source and timely onset: This figure illustrates the relationship between the bleeding source and the onset time. Understanding this relationship can help predict and manage PTPH, improving patient outcomes

As mentioned in the methods section, we defined four groups of bleeding sources. Figure 1. shows the onset of the bleeding in relation to the bleeding source. These findings appear to be statistically significant (p < 0.001). Bleeding due to erosion occurred on day in a median of postoperative day 15 (min. 5, max. 20), surgical bleedings on a median of day 1 after surgery (min. 0, max. 5), GI-bleedings on a median of day 20 after surgery (min. 8, max. 90) and diffuse bleedings on a median of day 2 after surgery (min. 0, max. 15).

Successive backward and forward multivariate testing did not lead to an applicable prediction model by means of combined risk factors neither for the parameter distribution over the sources nor the appearance of bleedings in general. Compared to the rest of the cohort, the cases with bleeding due to erosion only showed significant differences in the parameter of vessel invasion (p = 0.04). For the surgical bleedings, the differences persisted in kidney failure (p = 0.002), liver insufficiency (p = 0.005), the occurrence of embolism or thrombosis (p = 0.045) and the distribution of Clavien/Dindo classification towards major complication levels (p < 0.001). In the group developing GI bleeding, the distribution of the underlying diagnosis differed significantly from the overall cohort (p = 0.008). More neuroendocrine tumours (NETs) were found in this group. The number of patients needing antihypertensive medication was also considerably higher in this group (p = 0.016). Regarding the diffuse bleeding, preoperative anticoagulation showed a significant difference (p < 0.001) as well as liver insufficiency (p = 0.039). The other nominal and ordinal scaled parameters did not show significant findings for each entity. Comparing the differences of the interval scaled data over the bleeding sources, only the thrombocyte count on postoperative days one (p = 0.043), three (p = 0.015), and five differ significantly with the lowest count in the group developing surgical bleedings.

**Fig. 2:**
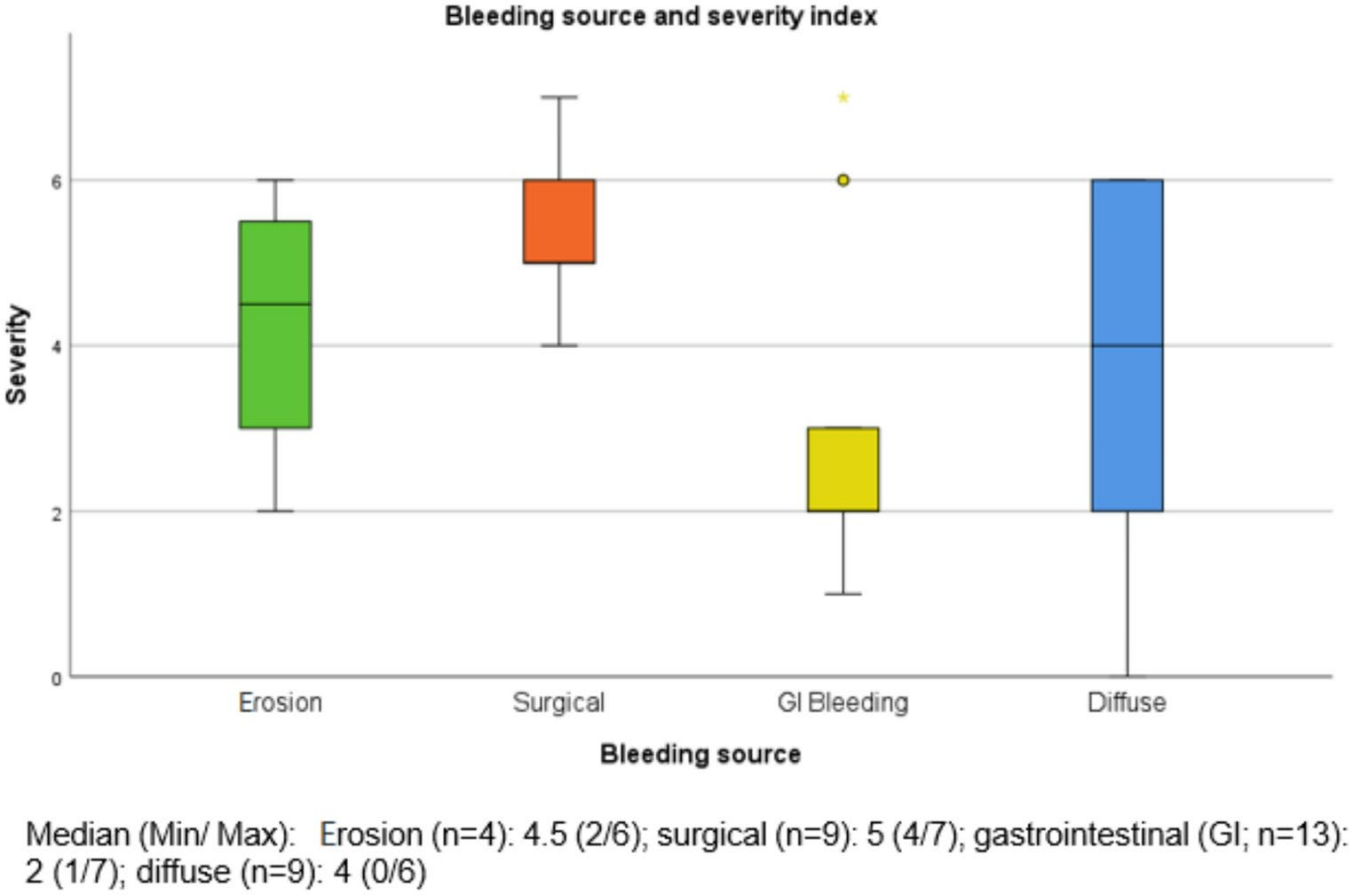
Bleeding source and severity index: This figure illustrates the relationship between the bleeding source and our newly defined severity index

Figure 2. shows the severity index (comp. methods section) in relation to the bleeding source. These findings appear to be statistically significant (p = 0.037).

## Discussion

The ISGPS definition is based on three criteria: time of onset, location and cause and severity.

### Implementing a new scoring and classification

Early onset is defined as bleeding within 24 h after the initial surgery, while late onset encompasses bleeding beyond this period. Alternative definitions, ranging from suggestions of a 5 to 30-day cut-off to differentiate early from late-onset PPH, are less commonly employed in clinical and scientific contexts. The timely onset indeed correlates with the bleeding source, e.g., surgical bleedings occurring with timely proximity to the initial surgery and bleeding due to erosion having a timely gap. Consequently, the importance of the timing of onset as an independent parameter is debatable, raising questions about its necessity and potential redundancy.

The second criterion from the ISGPS classification pertains to the location and cause of the bleeding. The description and identification of the bleeding source and its timing offer a more pragmatic and meaningful parameter than timing alone. This approach is advantageous for both research and clinical practice. It provides a clearer understanding of bleeding sources and their relationship to the timing of the bleed feasible for the PTPH as a newly defined complication entity following total pancreatectomy. There was a correlation between the onset, bleeding source, and severity index, leading to the third criterion.

### Severity of bleeding

The third criterion involves the severity of the bleeding. The current classification system for PPH includes several descriptive factors, which can be abstract. To address this, we have developed a scoring system that evaluates various factors (e.g., organ failure and the need for intervention) to provide a more objective measure of severity. This scoring system enhances comparability between individual cases and cohorts by quantifying descriptive factors.

### Comparison to the PPH classification according to ISGPS

The therapeutic course of action can be determined more directly and efficiently when the bleeding source is more precisely integrated into the classification and contains exactly named subgroups. In the traditional ISGPS classification, the therapeutic implications cannot be directly inferred from categorizing bleedings as PPH A, B, or C. The ISGPS classification, in summary, applies to the PTPH, but precision can be enhanced with emphasis on the definition of specific bleeding sources requiring certain actions. It is a well-established tool for postpancreatectomy haemorrhage in general [10], where specific bleeding sources due to POPF cannot be as clearly defined as following total pancreatectomy. Duarte Garcés et al. aimed to reappraise the ISGPS classification for PPH with a similar concept, showing that PPH A and no PPH had comparable outcomes. However, PPH B, in their cohort, contained a heterogeneous group with different bleeding sources or causes [11]. Maccabe et al. reported inconsistencies regarding the use of the ISGPS classification and moreover the grading in literature [12]. A combination of the bleeding source and the severity index is our approach to the definition of PTPH, which appeared feasible for our cohort.

### General risk factors for PTPH

Bleeding complications following total pancreatectomy are a relevant topic and are related to high morbidity and mortality. Preexisting conditions such as a history of bleeding or the need for anticoagulation, as well as basic parameters like age or BMI, did not significantly influence the likelihood of postoperative bleeding complications in our cohort. Robins et al. observed an increased rate of gastrointestinal bleeding in their study of patients undergoing total pancreatectomy and islet transplantation for chronic pancreatitis. Although their cohort is not directly comparable to ours, they similarly excluded anticoagulation and alcohol abuse as risk factors [13]. Portal-vein and other vascular reconstructions, although requiring therapeutic anticoagulation in the further course, did not correlate significantly with bleeding events. Previous studies have proven their feasibility and safety as options for resectioning expansive or infiltrating specimen [14, 15]. The cohort size somewhat explains that multivariate testing in the overall cohort did not lead to a favorable prediction model.

The identified correlations in the subgroups identified with univariate analyses regarding predictive factors also need more exploration in a larger cohort. That the group developing surgical bleeding has a higher Clavien/Dindo classification and is strongly related to organ failure corresponds with the findings of the correlation between bleeding source and our severity index. Although this analysis does not provide a separate multivariate model for each bleeding cause, the subdivision into bleeding sources remains a valuable specification for both research and clinical practice.

### Surgical and erosive bleeding – better known entities in pancreatic surgery

The source of surgical bleeding is well-defined and easily explained, although it was not predictable using pre-existing conditions within our cohort. A similar observation applies to erosive bleeds. The insufficiency of the hepaticojejunostomy or gastroenterostomy and the development of intra-abdominal abscesses represent an identifiable and understandable source of bleeding. Due to the small size of the subgroup, no predictable factors were apparent in our study. However, several factors are described in the literature as risk factors for insufficiency of hepaticojejunostomy or anastomotic leakage in general [16, 17].

### Gastrointestinal and diffuse bleeding – signs of coagulopathy?

The two remaining entities, gastrointestinal and diffuse bleeding, present more significant challenges in determining their underlying causes. Both could theoretically suggest a transient coagulopathy, although this was not explicitly reflected in the routine diagnostic evaluations retrospectively analyzed in this study. Notably, diffuse bleeding was the only source correlated with preoperative anticoagulation, indicating that anticoagulation may be a potential risk factor for this type of bleeding. GI-bleeding with late onset is described with an incidence of 2–18% following pancreatic resections [18]. Our incidence of 6.7% lies within this range. Understanding the mechanisms and their difference to those following e.g. pancreatic head resections should be the topic of further research. The role of the tumor stage remains unclear, as our analysis outlines an association between lymphatic and vessel involvement and bleeding complications with a higher bleeding prevalence in patients with vessel and lymphatic invasion and less bleeding complications in patients with a higher grading. However, the relation between the tumour-micro-environment and bleeding complications cannot sufficiently be explained tih the data provided.

However, it is well known that pancreatic adenocarcinoma has the highest cancer-associated risk for venous thrombose and consecutive embolism [19]. The pancreatic tissue is involved in the plasminogen cascade, and its complete removal might have a hypothetical influence on the cascade. Taking these findings into account, further analyses are mandatory as the current data does not allow to draw any more conclusions. Recent research discovered that plasminogen activators play a role in tumor invasion in pancreatic adenocarcinoma [20], but their role and specific relevance in the coagulation cascade is still unclear. Significant correlation of bleeding complications and embolisms in our cohort might also relate to these findings. Given the descriptive nature of the present data, it is not possible to draw further conclusions regarding the influence of tumour stage, vessel invasion or grading within this context.

### Limitations

We acknowledge key limitations of this study. The retrospective design and small subgroup sizes introduce biases and preclude comprehensive multivariate analyses of predictive factors.

## Conclusion

While specific risk factors for PTPH were not identified in our study, we have provided a practical and effective classification. This classification, which follows the ISGPS model but focuses on total pancreatectomies, the bleeding source, and clinical severity, is feasible for clinical practice and research. It thereby defines the entity of PTPH, providing a valuable contribution to pancreatic surgery.

## Supplementary Information

Below is the link to the electronic supplementary material.

Supplementary file1 (DOCX 55.6 KB)

## Data Availability

No datasets were generated or analysed during the current study.

## Declarations

### Submission declaration

This article has not been published elsewehere and is not in consideration for publication elsewhere.

### Other

Dr. med. Lea Timmermann is research associate at the cluster of excellence (Matters of Activity, Image Space Material), Humboldt University, Berlin.
