# Supplementary material for: Post-Total-Pancreatectomy-Hemorrhage (PTPH) – approaching a new definition
Source: Langenbecks Arch Surg. 2025 Sep 23;410(1):274. doi: 10.1007/s00423-025-03869-4 (PMC12454621; doi:10.1007/s00423-025-03869-4)
Supplement: Supplementary file 1 — (DOCX 55.6 KB) [file 423_2025_3869_MOESM1_ESM.docx]

Supplementary material

1. *Subgroup with no insufficiency of hepaticojejunostomy
   1.1. Demographics*

|  | All patients (n=175) | Bleeding complications (N=28) | No bleeding complications (N=147) | p-value |
| --- | --- | --- | --- | --- |
| Age (years) |  |  |  | 0.754 |
| Minimum | 34 | 38 | 34 |  |
| Maximum | 86 | 82 | 86 |  |
| Median | 68 | 69.5 | 67 |  |
|  |  |  |  |  |
| Sex N (%) |  |  |  | 0.291 |
| Female | 72 (41) | 9 (32) | 63 (42) |  |
| Male | 103 (59) | 19 (68) | 84 (57) |  |
|  |  |  |  |  |
| BMI (kg/m²) |  |  |  | 0.271 |
| Minimum | 17.2 | 17.7 | 17.2 |  |
| Maximum | 42.8 | 32.5 | 42.8 |  |
| Median | 24.45 | 24.1 | 24.5 |  |
|  |  |  |  |  |
| ASA N (%) |  |  |  | 0.81 |
| 1 | 4 (2) | 1 (4) | 3 (2) |  |
| 2 | 58 (35) | 8 (31) | 50 (36) |  |
| 3 | 98 (59) | 16 (62) | 82 (59) |  |
| 4 | 6 (4) | 1 (4) | 5 (4) |  |
|  |  |  |  |  |
| Malignancy N (%) | 140 (80) | 21 (75) | 119 (81) | 0.47 |
|  |  |  |  |  |
| Diagnosis N (%) |  |  |  | 0.595 |
| PDAC | 120 (69) | 18 (64) | 102 (69) |  |
| Cholangiocarcinoma | 10 (6) | 1 (4) | 9 (6) |  |
| NET | 4 (2) | 1 (4) | 3 (2) |  |
| Chronic pancreatitis | 21 (12) | 3 (11) | 18 (12) |  |
| IPMN | 11 (6) | 4 (14) | 7 (5) |  |
| Other malignant | 6 (3) | 1 (4) | 5 (3) |  |
| Other benign | 3 (2) | 0 (0) | 3 (2) |  |
|  |  |  |  |  |
| NAC in cases of underlying malignancy N (%) | 22 (15) | 3 (14) | 19 (15) | 0.914 |
|  |  |  |  |  |
| T state N (%) |  |  |  | 0.122 |
| 1 | 12 (9) | 1 (5) | 11 (10) |  |
| 2 | 43 (33) | 5 (26) | 38 (34) |  |
| 3 | 70 (53) | 10 (53) | 60 (54) |  |
| 4 | 6 (5) | 3 (16) | 3 (3) |  |
|  |  |  |  |  |
| N state N (%) |  |  |  | 0.147 |
| 0 | 41 (31) | 5 (26) | 36 (32) |  |
| 1 | 58 (44) | 6 (32) | 52 (46) |  |
| 2 | 32 (24) | 8 (42) | 24 (21) |  |
|  |  |  |  |  |
| Grading G (%) |  |  |  | 0.164 |
| 1 | 4 (4) | 0 (0) | 4 (4) |  |
| 2 | 67 (59) | 13 (81) | 54 (55) |  |
| 3 | 43 (38) | 3 (19) | 40 (41) |  |
|  |  |  |  |  |
| Resection state N (%) |  |  |  | 0.286 |
| 0 | 80 (64) | 10 (53) | 70 (65) |  |
| 1 | 46 (37) | 9 (47) | 37 (35) |  |
|  |  |  |  |  |
| L state N (%) |  |  |  | 0.069 |
| 0 | 94 (75) | 11 (58) | 83 (78) |  |
| 1 | 32 (25) | 8 (42) | 24 (22) |  |
|  |  |  |  |  |
| V state N (%) |  |  |  | 0.053 |
| 0 | 110 (87) | 14 (74) | 96 (90) |  |
| 1 | 16 (13) | 8 (26) | 11 (10) |  |
|  |  |  |  |  |
| Tumour size (mm) |  |  |  | 0.211 |
| Minimum | 4 | 17 | 4 |  |
| Maximum | 110 | 110 | 85 |  |
| Median | 32.5 | 35 | 32 |  |

- 1. *History of preexisting conditions*

|  | All patients (n=175) | Bleeding complications (N=28) | No bleeding complications (N=147) |  |
| --- | --- | --- | --- | --- |
| Insulin dependent diabetes mellitus N (%) | 45 (26) | 10 (36) | 35 (24) | 0.187 |
| Medication for arterial hypertonus N (%) | 92 (53) | 17 (61) | 75 (51) | 0.346 |
|  |  |  |  |  |
| History of thrombosis/ embolism N (%) |  |  |  | 0.516 |
| Venous thrombosis | 5 (3) | 0 (0) | 5 (3) |  |
| Lung artery embolism | 6 (3) | 0 (0) | 6 (4) |  |
| Coronary heart disease/ pAVK | 13 (7) | 3 (11) | 10 (7) |  |
| Ischemic stroke | 3 (2) | 1 (4) | 2 (1) |  |
|  |  |  |  |  |
| History of bleeding N (%) | 4 (2) | 0 (0) | 4 (3) | 0.377 |
| History of liver disease N (%) | 13 (7) | 2 (7) | 11 (8) | 0.950 |
| History of alcohol abuse N (%) | 18 (10) | 2 (7) | 16 (11) | 0.55 |
|  |  |  |  |  |
| Preoperative anticoagulation N (%) |  |  |  | 0.59 |
| Platelet aggregation inhibitor | 21 (12) | 4 (14) | 17 (12) |  |
| Coumarine derivatives | 3 (2) | 1 (4) | 2 (1) |  |
| Other oral anticoagulation | 5 (3) | 1 (4) | 4 (3) |  |
| Other | 2 (1) | 1 (4) | 1 (1) |  |

- 1. *Surgery*

| Surgical time (minutes) |  |  |  | 0.208 |
| --- | --- | --- | --- | --- |
| Minimum | 113 | 196 | 113 |  |
| Maximum | 828 | 613 | 828 |  |
| Median | 336 | 351.5 | 334 |  |
|  |  |  |  |  |
| Access N (%) |  |  |  | 0.817 |
| Open | 166 (95) | 27 (96) | 139 (95) |  |
| Robotic assisted | 7 (4) | 1 (4) | 6 (4) |  |
| Hybrid | 2 (1) | 0 (0) | 2 (1) |  |
|  |  |  |  |  |
| Splenectomy N (%) | 86 (49) | 14 (50) | 72 (49) | 0.921 |
|  |  |  |  |  |
| Portal vein resection/ reconstruction N (%) | 15 (9) | 2 (7) | 13 (9) | 0.768 |
| Other vascular resections/ reconstructions | 10 (6) | 1 (4) | 9 (6) | 0.594 |
|  |  |  |  |  |
| Intraoperative blood transfusion (bags) |  |  |  | 0.093 |
| Minimum | 0 | 0 | 0 |  |
| Maximum | 11 | 11 | 8 |  |
| Median | 0 | 0 | 0 |  |
|  |  |  |  |  |
| Intraoperative fresh frozen plasma (bags) |  |  |  | 0.059 |
| Minimum | 0 | 0 | 0 |  |
| Maximum | 29 | 29 | 22 |  |
| Median | 0 | 0 | 0 |  |

- 1. *Laboratory testing*

|  | All patients (n=175) | Bleeding complications (N=28) | No bleeding complications (N=147) | p-value |
| --- | --- | --- | --- | --- |
| Preoperative prothrombin time (%) |  |  |  | 0.253 |
| Minimum | 58 | 66 | 58 |  |
| Maximum | 128 | 115 | 128 |  |
| Median | 94.5 | 91 | 96 |  |
|  |  |  |  |  |
| Postoperative prothrombin time (%) |  |  |  | 0.112 |
| Minimum | 45 | 45 | 49 |  |
| Maximum | 111 | 100 | 111 |  |
| Median | 83 | 78 | 84 |  |
|  |  |  |  |  |
| Prothrombin time POD 1 (%) |  |  |  | 0.617 |
| Minimum | 22 | 55 | 22 |  |
| Maximum | 108 | 87 | 108 |  |
| Median | 71 | 70 | 71 |  |
|  |  |  |  |  |
| Prothrombin time POD 3 (%) |  |  |  | 0.804 |
| Minimum | 19 | 47 | 19 |  |
| Maximum | 127 | 103 | 127 |  |
| Median | 72 | 70 | 72 |  |
|  |  |  |  |  |
| Prothrombin time POD 5 (%) |  |  |  | 0.668 |
| Minimum | 38 | 52 | 38 |  |
| Maximum | 111 | 100 | 111 |  |
| Median | 77 | 74 | 77 |  |
|  |  |  |  |  |
| Preoperative activated partial thromboplastin time (sec) |  |  |  | 0.803 |
| Minimum | 23.2 | 25.5 | 23.1 |  |
| Maximum | 70.5 | 58.4 | 70.5 |  |
| Median | 33 | 32.5 | 33.1 |  |
|  |  |  |  |  |
| Postoperative activated partial thromboplastin time (sec) |  |  |  | 0.139 |
| Minimum | 20.5 | 26.2 | 20.5 |  |
| Maximum | 207.4 | 207.4 | 54.4 |  |
| Median | 31 | 32.3 | 30.8 |  |
|  |  |  |  |  |
| Activated partial thromboplastin time POD 1 (sec) |  |  |  | 0.034 |
| Minimum | 21.7 | 29.7 | 21.7 |  |
| Maximum | 121.5 | 121.5 | 78.1 |  |
| Median | 37.6 | 38.6 | 37.3 |  |
|  |  |  |  |  |
| Activated partial thromboplastin time POD 3 (sec) |  |  |  | 0.117 |
| Minimum | 21.2 | 31.5 | 21.2 |  |
| Maximum | 164.7 | 164.7 | 85.2 |  |
| Median | 38.8 | 42.1 | 38.4 |  |
|  |  |  |  |  |
| Activated partial thromboplastin time POD 5 (sec) |  |  |  | 0.596 |
| Minimum | 21 | 28.6 | 21 |  |
| Maximum | 246 | 57.9 | 246 |  |
| Median | 34.25 | 36.7 | 33.6 |  |
|  |  |  |  |  |
| Preoperative platelet count (/nl) |  |  |  | 0.937 |
| Minimum | 112 | 116 | 112 |  |
| Maximum | 674 | 545 | 674 |  |
| Median | 251.5 | 254.5 | 251.5 |  |
|  |  |  |  |  |
| Postoperative platelet count (/nl) |  |  |  | 0.128 |
| Minimum | 19 | 19 | 57 |  |
| Maximum | 629 | 448 | 629 |  |
| Median | 207 | 184.5 | 214 |  |
|  |  |  |  |  |
| Platelet count POD 1 (/nl) |  |  |  | 0.005 |
| Minimum | 51 | 51 | 72 |  |
| Maximum | 516 | 369 | 516 |  |
| Median | 191.5 | 154 | 201 |  |
|  |  |  |  |  |
| Platelet count POD 3 (/nl) |  |  |  | 0.012 |
| Minimum | 30 | 30 | 64 |  |
| Maximum | 687 | 385 | 687 |  |
| Median | 190.5 | 135.5 | 197 |  |
|  |  |  |  |  |
| Platelet count POD 5 (/nl) |  |  |  | 0.033 |
| Minimum | 57 | 57 | 67 |  |
| Maximum | 907 | 608 | 907 |  |
| Median | 256 | 170 | 262.5 |  |
|  |  |  |  |  |
| Postoperative antithrombin 3 (%) |  |  |  | 0.876 |
| Minimum | 45 | 51 | 45 |  |
| Maximum | 119 | 119 | 114 |  |
| Median | 81 | 81 | 81 |  |
|  |  |  |  |  |
| Antithrombin 3 POD 1 (%) |  |  |  | 0.936 |
| Minimum | 6 | 57 | 6 |  |
| Maximum | 120 | 110 | 120 |  |
| Median | 81.5 | 80.5 | 81.5 |  |
|  |  |  |  |  |
| Postoperative fibrinogen (g/l) |  |  |  | 0.143 |
| Minimum | 1.72 | 1.72 | 1.86 |  |
| Maximum | 6.42 | 6.02 | 6.42 |  |
| Median | 3.41 | 3.17 | 3.75 |  |
|  |  |  |  |  |
| Preoperative CA 19.9 (kU/l) |  |  |  | 0.652 |
| Minimum | 1.7 | 2 | 1.7 |  |
| Maximum | 6582 | 6582 | 5711 |  |
| Median | 140 | 16.7 | 153 |  |
|  |  |  |  |  |
| Preoperative CEA (µg/l) |  |  |  | 0.722 |
| Minimum | 0.7 | 2.2 | 0.7 |  |
| Maximum | 78.7 | 22.5 | 78.7 |  |
| Median | 3.4 | 5.8 | 3.2 |  |

- 1. *Perioperative course and complications*

|  | All patients (n=175) | Bleeding complications (N=28) | No bleeding complications (N=147) | p-value |
| --- | --- | --- | --- | --- |
| Inhospital stay (d) |  |  |  | 0.002 |
| Minimum | 1 | 4 | 1 |  |
| Maximum | 87 | 66 | 87 |  |
| Median | 16 | 24 | 14 |  |
|  |  |  |  |  |
| ICU stay (d) |  |  |  | 0.027 |
| Minimum | 0 | 2 | 0 |  |
| Maximum | 203 | 49 | 203 |  |
| Median | 4 | 9.5 | 4 |  |
|  |  |  |  |  |
| Overall complications N (%) | 107 (61) | 28 (100) | 79 (54) | <0.001 |
|  |  |  |  |  |
| Kidney failure | 31 (18) | 12 (43) | 19 (13) | <0.001 |
| Liver failure | 13 (7) | 7 (25) | 6 (4) | <0.001 |
|  |  |  |  |  |
| SSI N (%) | 32 (18) | 7 (25) | 25 (17) | 0.316 |
| DGE N (%) | 24 (14) | 3 (11) | 21 (14) | 0.615 |
|  |  |  |  |  |
| Reoperation N (%) | 28 (16) | 15 (54) | 13 (9) | <0.001 |
|  |  |  |  |  |
| 30-day-mortaility N (%) | 8 (5) | 4 (14) | 4 (3) | 0.008 |
|  |  |  |  |  |
| Readmission rate N (%) | 20 (12) | 8 (29) | 12 (8) | 0.002 |
|  |  |  |  |  |
| Clavien/ Dindo N (%) |  |  |  | <0.001 |
| 1 | 5 (3) | 1 (4) | 4 (3) |  |
| 2 | 39 (22) | 2 (7) | 37 (25) |  |
| 3a | 13 (7) | 6 (21) | 7 (5) |  |
| 3b | 18 (10) | 5 (18) | 13 (9) |  |
| 4a | 15 (9) | 5 (18) | 10 (7) |  |
| 4b | 2 (1) | 2 (7) | 0 (0) |  |
| 5 | 17 (10) | 7 (25) | 10 (7) |  |
|  |  |  |  |  |
| Thrombosis/ embolism during inhospital stay N (%) | 12 (7) | 6 (21) | 6 (4) | <0.001 |
